# Isolation and Identification Antagonistic Bacterium *Paenibacillus tianmuensis* YM002 against *Acidovorax citrulli*

**DOI:** 10.3389/fpls.2023.1173695

**Published:** 2023-06-12

**Authors:** Young Mo Koo, A Yeong Heo, Hyong Woo Choi

**Affiliations:** ^1^ Department of Plant Medicals, College of Life Sciences and Biotechnology, Andong National University, Andong, Republic of Korea; ^2^ Institute of Cannabis Biotechnology, Andong National University, Andong, Republic of Korea

**Keywords:** Paenibacillus tianmuensis, PGPR, cucumber, induced resistance, biological control

## Abstract

In this study, we aimed to screen antagonistic microorganisms against *Acidovorax citrulli*, the causal agent of bacterial fruit blotch, which is known to induce sever diseases in cucurbit crops. From 240 bacterial strains isolated, only one unknown bacterial isolate, named YM002, showed significant antagonistic activity against *A*. *citrulli* KACC17909. Further experiments revealed that YM002 shows antagonistic activity against all tested *A*. *citrulli* strains, including KACC17000, KACC17001 and KACC17005, to different degrees. The phylogenetic analysis of 16S rRNA sequences identified YM002 as *Paenibacillus tianmuensis*. Importantly, pretreatment of cucumber (*Cucumis sativus*) leaves with YM002 enhanced disease resistance as observed by significantly reduced necrotic symptom development and bacterial growth. YM002-induced resistance accompanied by enhanced expression of defense-related genes, such as *PAL1*, *PR1-1a* and *CTR1*. Importantly, culture filtrate of YM002 significantly suppressed biofilm formation and swimming motility of *A. citrulli*, which is indispensable for its full virulence. In addition to its antagonistic activity, YM002 showed a various plant growth promotion (PGP)-related traits, such as production of ammonia production, amylase production, ACC deaminase production, inodole-3-acetic acid production, extracellular protease production, siderophore production, and zinc solubilization activities. Indeed, treatment of cucumber roots with YM002 significantly enhanced plant growth parameters, such as fresh and dry weight of leaves or roots. This study suggests the potential of YM002 as an effective PGPR with biological control activity against *Acidovorax citrulli* in cucumber plants.

## 1 Introduction

In natural conditions, various rhizosphere microorganisms exist in the soil while interacting with plants. In the rhizosphere soil of plants, there are up to 10^11^ microbial cells (Egamberdieva et al., 2008) and more than 30,000 different *prokaryotes* are exist per gram of root (Mendes et al., 2011). The optimal plant rhizosphere microbiome not only reduces the incidence and/or severity of plant diseases by protecting plants from different pathogens (Bloemberg and Lugtenberg, 2001), but also promotes plant growth *via* enhancing the plant mineral nutrition and regulating the production of plant growth (Bloemberg and Lugtenberg, 2001; Heo et al., 2022; Kapadia et al., 2022; Kong and Liu, 2022). Thus it is important to understand and identify the beneficial microorganisms for the development of a sustainable agricultural system.

*Acidovorax citrulli*, previously known as *Acidovorax avenae* subsp. *citrulli*, is the causal agent of bacterial fruit blotch (BFB) of cucurbits (Song et al., 2020; Kim et al., 2021; Lee et al., 2021; Rahimi-Midani et al., 2021). *A*. *citrulli* is a gram-negative bacteria and is known to be transmitted through seed. Although the timing of symptom onset can vary according to environmental conditions, BFB symptoms can emerge within 6-10 days after germination when infested seeds are directly seeded. Subsequently, the bacteria from the infected plants can be rapidly transmitted to healthy seedlings during overhead irrigation. Under favorable environmental conditions (high humidity and temperature), *A*. *citrulli* can induce more rapid BFB symptom development (Burdman et al., 2005; Burdman and Walcott, 2012). Initial symptoms of infested seedlings are observed as water-soaked lesions on cotyledons (Schaad et al., 2003). Then, the whole plant shows severe wilting symptoms and eventual death. In the case of fruits, BFB induces black-colored and water-soaked lesions, resulting in rot and disintegration of the flesh (Latin and Hopkins, 1995). *A. citrulli* occurs worldwide and destructive disease of cucurbit crops (Schaad et al., 1978; Willems et al., 1992; Schaad et al., 2008). Since the first outbreak of BFB was observed at the Mariana Islands in 1987 on commercial watermelon (*Citrullus lanatus*) fields (Wall and Santos, 1988), it continues to cause disease on different cucurbits, such as honeydew melon (*Cucumis melo*) (Isakeit et al., 1997), pumpkin (*Cucurbita pepo*) (Walcott et al., 2004), squash (*Cucurbita maxima*) (Walcott et al., 2004), cucumber (*Cucumis sativus*) (Martin et al., 1999) and watermelon (Burdman et al., 2005). *A. citrulli* strains are divided into two groups based on molecular analysis, such as DNA fingerprinting and multilocus sequence typing (MLST) analysis of housekeeping genes, and fatty acid methyl ester profiles (Walcott et al., 2000; Walcott et al., 2004; Burdman et al., 2005; Feng et al., 2009). Group I mainly consists of strains isolated from melons and other cucurbit crops, except for watermelon, and causes significant damage to various cucurbit crops. In contrast, group II strains are mainly isolated from watermelons and are highly pathogenic to watermelons, but less damaging to other cucurbits and crops (Silva et al., 2016; Zivanovic and Walcott, 2017).

Plant growth-promoting rhizobacteria (PGPR) are symbiotic with plants and provide various benefits related to plant growth. In terms of nutrient acquisition and plant growth benefits, PGPR enhances nutrient uptake by converting high molecular weight insoluble mineral components of the soil into a low molecular weight form that can be used by plants. For example, siderophore production, zinc solubilization and phosphate solubilization activities of PGPR can improve the root system structure of host plants by improving water and mineral retrieval ability. Nitrogen fixation by PGPR converts nitrogen gas from the air into nitrogenous organic compounds, a form that plants can absorb (Richardson and Simpson, 2011; Backer et al., 2018; Trivedi et al., 2020). The PGPR can stimulate plant growth by metabolizing small molecules in plants to modulate phytohormones such as indole-3-acetic acid (IAA), gibberellin, ethylene, cytokinins, and abscisic acid (Rehman et al., 2020). The PGPR uses L-tryptophan to produce IAA, which plays an important role in the promotion of plant growth and physiological processes such as embryogenesis, organogenesis, vascular differentiation, root and shoot development, trophic growth, and fruit development (Etchells et al., 2016).

PGPR can enhance plant disease resistance by directly suppressing pathogen growth *via* (i) production of hydrolytic enzymes, antimicrobial compounds, and biosurfactants, (ii) hyper-parasitism, (iii) competitive inhibition, etc. (Zehra et al., 2021; Bonaterra et al., 2022; Choi and Ahsan, 2022). Especially, the genus *Paenibacillus* spp. found in various environmental conditions and produce a variety of antibacterial compounds, including lantibiotics, polyketides, lipopeptides, macrolide, and peptide–polyketide (Wu et al., 2022). On the other hands, PGPR can also indirectly enhance disease resistance by activating induced resistance (IR) that involves priming states of plants (Bonaterra et al., 2022). Different external stimuli, such as pathogens, beneficial microbes, and chemicals cues, are known to trigger the establishment of priming by acting as warning signals (Mauch-Mani et al., 2017). Primed plants exhibit robust and enhanced activation of defense-related gene expression upon pathogen infection, there by exhibiting enhanced resistance to different pathogens. In cucumber (*Cucumis sativus* L.) plants, nonpathogenic *Fusarium oxysporum* CS-20 triggered IR against pathogenic *F*. *oxysporum* f. sp. *cucumerinum*, a causal agent of Fusarium wilt (Pu et al., 2014). *F. oxysporum* CS-20-induced IR accompanied by enhanced expression of *PR1*, *PR3*, *LOX1* and *PAL1*. Similarly, treatment of cucumber plants with IR-inducing fungi, *Trichoderma harzianum* Tr6, enhanced expression of *PR1*, *PAL1* and *LOX1* (Alizadeh et al., 2013). Thus utilization of beneficial microbes, like PGPR, becomes more and more important to for sustainable agriculture system, which requires environmentally friendly measures that can alternate chemical pesticides.

This study aims to identify and isolate antagonistic microorganisms against *A*. *citrulli* from soil sample, and verify their disease control and PGP activities *in vitro* and *in vivo*. From the screening, we isolated and identified *Paenibacillus tianmuensis* strain YM002 (YM002) from the soil as an antagonistic microorganism against *A. citrulli*. Cell suspension or culture filtrate of YM002 significantly enhanced the resistance of cucumber against *A. citrulli* through its direct antimicrobial activity and indirect IR-inducing activity. Moreover, YM002 showed various PGPR-related traits and enhanced the growth of cucumber plants. Our findings suggest that YM002 can be used as a potential biocontrol agent against *A. citrulli* and bio-fertilizer of cucumber plants.

## 2 Materials and methods

### 2.1 Soil samples collection and bacteria isolation

Isolation of antagonistic bacteria was performed by serial dilution method as previously described (Heo et al., 2022). Briefly, rhizosphere soil samples of various plants were collected from Agi mountain, Andong, Republic of Korea (36°32’22.9”N 128°56’14.3”E). One gram of samples was dissolved in 10 ml of sterilized water and mixed by using a shaker (DLAB Scientific co., Ltd., China) at room temperate for 2 h. The mixture was filtered using cheese cloths. One milliliter of the mixture was transferred sterilized 1.5 ml micro-tube and serially diluted up to 10^-7^. Subsequently, 0.1 ml of the diluted suspension was seeded on King’s B agar plates (KB, MB cell, Korea), then incubated at 28 °C for 48 h. All single colonies were transferred to fresh KB broth using sterile toothpicks.

### 2.2 Screening of antagonistic bacteria by agar plate assay

*Acidovorax citrulli* strains KACC17909, KACC17000, KACC17001, and KACC17005 were generously provided by Korean Agricultural Culture Collection (KACC; http://genebank.rda.go.kr/). Antagonistic activity of unknown bacterial isolates against *Acidovorax citrulli* was screened as previously described with minor modifications (Bauer et al., 1966). Briefly, each bacterial isolate was grown in a KB broth medium at 180rpm 28 °C for 72 h. To prepare the agar plug, 100 μl of bacterial suspension was spread on KB agar plates and incubated at 28°C for 72h. After incubation, plates were punctured with a sterile cork borer (5.5 mm in diameter). Agar plugs of bacterial isolates were placed in the middle of KB agar plates seeded with *A. citrulli* strain KACC17909. Among 240 unknown bacteria isolated and tested, only 1 bacterial isolate named YM002 showed significant antagonistic activity against *A*. *citrulli* KACC17909. After the screening, antagonistic activity of YM002 is further tested against another *A. citrulli* strains KACC17000, KACC17001, and KACC17005. The plates were incubated at 28°C for 72h and the clear zone (total inhibition zone diameter – YM002 growth diameter) was measured. Swimming motility of YM002 was also measured by colony diameter during the agar plate assay in the presence of different strains of *A. citrulli*. All bacterial isolates were preserved in 20% glycerol (v/v) at -80°C. The strain YM002 was deposited to the KACC (accession number: KACC92433P).

### 2.3 Identification of antagonistic bacteria

To identify bacterial strain showing antagonistic activity against *A. citrulli*, 16s rRNA sequencing and phylogenetic analysis were performed. Genomic DNA was extracted by using a Genomic DNA prep kit (BIOFACT, Daejeon, Korea), according to the manufacturer’s instructions. 16s rRNA region was amplified using a universal primer set, 27F (5′-AGA GTT TGA TCC TGG CTC AG-3′) and 1492R (5′-GGT TAC CTT GTT ACG ACT T-3′) (Lane, 1991; Weisburg et al., 1991). PCR reaction was performed with a mixture containing 0.5 μl Pfu-X (2.5 U/μl), 5 μl 10X Pfu-X reaction buffer, 1 μl 10 mM dNTP mix, 2 μl DNA template, and 2 μl of each primer. The PCR cycle was performed at an initial denaturation (2 minutes at 95 °C) and followed 33 cycles with denaturation at 95 °C for 30 seconds, annealing at 56 °C for 30 seconds, extension at 72 °C for 30 seconds, and a final extension at 72 °C for 5 minutes. Amplified PCR product was purified using Gel & PCR purification system (BIOFACT, Daejeon, Korea), according to the manufacturer’s instructions. The 16s rRNA gene of antagonistic bacteria was sequenced by Solgent sequence analysis services (Solgent co., Ltd, Korea). The sequence data were aligned by using Lasergene sequence analysis software (Burland, 2000). The resulting sequence of the 16s rRNA gene was analyzed with NCBI’s Gen-Bank sequence database (http://www.ncbi.nlm,nih.gov) to identify the closest species relatives. The phylogenetic tree was constructed by Maximum likelihood methods with bootstrap values of 1,000 replications using MEGA X (Kumar et al., 2018).

### 2.4 PGP-related traits

The extracellular amylase production ability of YM002 was determined as previously described (Cappuccino and Sherman, 1996) with slight modifications. Briefly, YM002 agar plug was inoculated on the center of starch agar plates (5.0 g peptone, 3.0 g yeast extract, 2.0 g soluble starch, and 15.0 g agar/L, pH adjusted to 7.0) for 5 days at 28 °C. After incubation, 3% iodine solution was poured into the plates and incubated for 20 minutes at room temperature. The diameter of the yellow-colored clear zone was measured.

To measure the ACC deaminase activity, minimal Dworkin and Foster (DF) salt media was prepared as previously described (Penrose et al., 2001). Briefly, the trace elements (10mg H_3_BO_3_, 11.19mg MnSO_4_·H_2_O, 124.6mg ZnSO_4_.7H_2_O, 78.22mg CuSO_4_·5H_2_O, and 10mg MoO_3_) were dissolved in 100 ml sterile distilled water (DW). FeSO_4_ solution was prepared by dissolving 100 mg of FeSO_4_·7H_2_O in 10 ml sterile DW. Medium compounds (4.0g KH_2_PO_4_, 6.0g Na_2_HPO_4_, 0.2g MgSO_4_.7H_2_O, 2.0g glucose, 2.0g gluconic acid, 2.0g citric acid, and 15g agar) and 0.1 ml of each solution were dissolved in 1L DW (pH adjusted to 7.2) and autoclaved. After autoclave, 3.0 mM 1-aminocyclopropane-1-carboxylic acid (ACC) was supplemented as a sole nitrogen source. YM002 agar plug was inoculated on the center of minimal DF agar medium for 7 days at 28°C. After incubation, the colony diameter was measured.

The extracellular protease production of YM002 was determined by monitoring the clear zone formation on skim milk agar plates (Chu, 2007). Briefly, 10g of skim milk was dissolved in 400 ml DW, and 1g yeast extract and 15g agar were dissolved in 600 ml DW, separately. Each reagent was autoclaved and mixed on a clean bench. YM002 agar plug inoculated on the center of skim milk medium. YM002 was incubated for 5 days at 28°C, and the diameter of the clear zone was measured.

To check siderophore production activity, CAS agar plate assay was performed as previously described (Schwyn and Neilands, 1987; Arora and Verma, 2017). Briefly, 60.5mg chrome azurol S (CAS) was dissolved in 50 ml sterile deionized water and added to 40 ml hexadecyl trimethyl ammonium bromide (HDTMA). HDTM solution was prepared by dissolving 72.9 mg HDTMA in 40 ml sterile DW. Then, mixed 90 ml CAS-HDTMA solution with 900 ml NA agar medium. One millimolar ferric chloride solution was prepared by dissolving FeCl_3_·6H_2_O in 10 mM HCl. NA agar medium and Ferric chloride solution were autoclaved separately. After autoclaving, both reagents were mixed on a clean bench. YM002 agar plug inoculated on CAS agar plate for 7 days at 28°C. After incubation, the color change from blue to yellow was observed (Louden et al., 2011).

The zinc solubilization activity of YM002 was determined as previously described (Bapiri et al., 2012). PKV medium (10.0 g glucose, 1.0 g (NH_4_)SO_4_, 0.2 g KCl, 0.2 g K_2_HPO_4_, 0.1 g MgSO_4_, 0.2 g yeast extract and 15 g agar/L, pH 7.0) supplemented separately with either 1% ZnO or 1% ZnCO_3_. YM002 agar plug inoculated on the center of PKV agar medium for 5 days at 28°C. After incubation, the diameter of the clear zone was measured.

For ammonia (NH_3_) production assay, YM002 was grown in KB broth medium for 72 h and adjusted OD_600_ of 0.05 in sterile DW. One milliliter of cell suspension was inoculated at 95 ml of peptone water broth for 48 h at 48 °C. One milliliter of supernatant was mixed with 1 mL of Nessler’s reagent and 8.5 mL of sterile DW (Goswami et al., 2015). The mixture color change from brown to yellow indicates NH_3_ production, which is observed by absorbance at 450 nm by using a Nanoready Touch microvolume spectrophotometer (Genomic Base, Korea). The NH_3_ concentration was estimated using the standard curve generated by ammonia sulfate ranging from 1 to 10 μmol/mL. The experiment was performed with three replicates.

The quantification of indole-3-acetic acid (IAA) production was estimated using Salkowski’s reagent (Bric et al., 1991). YM002 was grown in KB broth medium for 72 h and adjusted to OD_600_ of 0.05 in sterile DW. One milliliter of YM002 cell suspension was inoculated into 95 ml of KB broth medium containing 3 mM L-tryptophan. The culture was incubated in a shaking incubator at 180 rpm at 28 °C for 72 h. One milliliter of supernatant was mixed with two milliliters of Salkowski’s reagent (0.5 M FeCl_3_·6H_2_O in 35% perchloric acid), then incubated at room temperature for 30 minutes under dark conditions. The mixture color change from brown to pink indicates IAA production, which is observed by absorbance at 530nm by using a Nanoready Touch microvolume spectrophotometer (Genomic Base, Korea). The standard curve of IAA concentration range from 10 to 100 μg/mL was prepared by using commercial IAA (Sigma, USA). The experiment was performed with three replicates.

### 2.5 Biofilm formation assay

Biofilm formation inhibitory activity of culture filtrate (CF) of YM002 against *A*. *citrulli* was measured as previously described (O’Toole et al., 1999; Masum et al., 2017). To prepare cell-free CF, YM002 was inoculated from a single colony into 100 ml of KB broth and incubated in a shaking incubator at 180 rpm at 28 °C for 72 h. Cultures were transferred to a 50 mL falcon tube and centrifuged at 10,000 rpm for 10 min. The supernatant was filtered through the membrane filter with a pore size of 0.22 μm (PALL Life Sciences, USA). One milliliter of *A*. *citrulli* strain KACC17001 (O.D_600_ = 0.05) was mixed with 9 mL of fresh KB broth with different concentrations of YM002 CF (1, 3 and 10%) with five replicate wells per treatment, then seeded in 6-well cell culture plate. Plates were incubated at 28 °C without agitation for 24 h and 48 h, then bacterial cells were carefully discarded. Ten milliliters of 0.1% crystal violet in sterile DW were added to each well for 30 minutes. The plates were then washed with sterile DW, air dried for 30 min, and resuspended in 10 mL of 95% ethanol for 20 minutes. The absorbance was measured at 590 nm.

### 2.6 Swimming motility assay

The effect of YM002 culture filtrate against swimming motility of *A*. *citrulli* was determined using methods previously described with slight modifications (Bahar et al., 2009; Masum et al., 2017). *A*. *citrulli* KACC17001 was grown in KB broth for 24 h, centrifuged at 10,000 rpm for 10 min, and the pellet was washed twice with sterile DW. The pellet was resuspension in sterile DW and its concentration was adjusted to OD_600_ of 0.05. Two microliters of bacterial suspension were inoculated on the center of a soft NA (0.3% agar) plate supplemented with 0, 25, and 50% YM002 CF. After incubating the plates for 48h at 28 °C, the colony diameter of *A*. *citrulli* was measured. This experiment was done with 8 replicates for each.

### 2.7 *In vivo* plant growth promoting activity

The plant growth promoting (PGP) activity of YM002 was tested by using root dipping methods (Orhan et al., 2006). Two-week-old cucumber (*Cucumis sativus* L. cv. Joeunbaekdadaki) roots were submerged into 10^5^ and 10^6^ cfu/mL of YM002 cell suspension for 30 min. Control cucumbers were dipped into sterile tap water. Cucumber plants treated with or without YM002 were transferred into the pot (10 × 9 cm), filled up with commercial potting soil, and their growth was monitored at 25 °C for 14 days. To measure the PGP activity of YM002, different growth parameters, including height, number of leaves, root length, and fresh and dry weights of leaves, stems, and roots were measured.

### 2.8 Biocontrol of *A*. *citrulli* by *Paneibacillus tianmuensis* YM002 in pot experiments

To test the biocontrol activity of YM002, cucumber plants were inoculated with spontaneous mutant of *A. citrulli* KACC17001 which shows Rifampicin resistance in the presence or absence of YM002 by using the spray inoculation methods (Song et al., 2020). To generate spontaneous mutant of *A. citrulli* KACC17001, 0.5 ml of 10^9^ cfu/ml of bacterial cell suspension was inoculated onto KB agar plate containing 10 µg/ml of rifampicin (Rif), and incubated at 28 °C for 3 days. The mutant colony survived on KB agar plate containing Rif was obtained and used for bacterial growth assay. YM002 was inoculated from a single colony into 100 ml of KB broth and incubated shaking incubator at 180 rpm at 28 °C for 72 h. Two-week-old cucumber plants were sprayed with either 50 ml of 10% CF of YM002 or 1 × 10^6^cfu/ml of cell suspension with 0.01% tween 20. Control plants were sprayed with sterile DW with 0.01% tween 20. After 24h, 50 ml of *A. citrulli* strain KACC 17000, 17001, 17005 and 17909 (1 × 10^7^ cfu/ml) were sprayed. Eight leaf discs per plant were collected by using a cork borer (diameter = 5.5 mm) for real-time qPCR and bacterial titer measurement. After 5 days of inoculation, disease severity was measured by monitoring the percentage of the necrotic area by using ImageJ software (Abramoff et al., 2004).

### 2.9 Real-time qPCR analysis

Defense-related gene expression was determined by using real-time quantitative polymerase chain reaction (RT-qPCR). The cucumber leaves were ground by using SILAMAT S6 (Ivoclar Vivadent Inc., Austria). Total RNA was extracted by using the APure™ Plant RNA extraction kit (Genomic Base, Korea). Complementary DNA was constructed by using BioFACT™ RT-Kit (BIOFACT Co., Ltd., Korea), according to the manufacturer’s instructions. RT-qPCR was performed in Linegene 9600 Fluorescent Quantitative detection system (Bioer Technology, China). A reaction mixture of 20 μl was prepared by mixing 10 μl SYBR Green Real Time PCR master Mix (TOYOBO CO., LTD., Japan) with 2 μl cDNA (1:20 diluted form), 6.4 μl sterilized distilled water and 0.8 μl of 10 pmole primers. *Actin* is used as a reference gene to normalize the expression of *CONSTITUTIVE TRIPLE RESPONSE 1* (*CTR1*), *Pathogenesis-related protein 1-1a* (*PR1-1a*), *Phenylalanine ammonia-lyase 1* (*PAL1*), *Ascorbate peroxidase* (*APOX*) and *Lipoxygenase 1* (*LOX1*) in RT-qPCR. Primers used in this experiments are provided in **Supplementary Table 1**. The RT-qPCR cycle was performed at an initial denaturation (20 second at 95 °C), 40 cycles of denaturation (95 °C for 20 sec), annealing (56 °C for 20 sec) and extension (72 °C for 20 sec), and followed final extension (72 °C for 2 min). The RT-qPCR data were calculated by using the 2^− ΔΔCt^ method. All RT-qPCR experiment was performed with 3 replicates for each sample.

### 2.10 Statistical analysis

For all experiments, analysis of variance (ANOVA) and Duncan’s multiple range test were performed by using IBM SPSS version 28 software (SPSS Inc., USA). Data were expressed as mean ± standard deviation (SD) from triplicates of each experiment. Significant differences were considered at *P* ≤ 0.05.

## 3 Results

### 3.1 Isolation and identification of antagonistic bacterium *Paneibacillus tianmuensis* YM002 against *Acidovorax citrulli*

**Figure 1:**
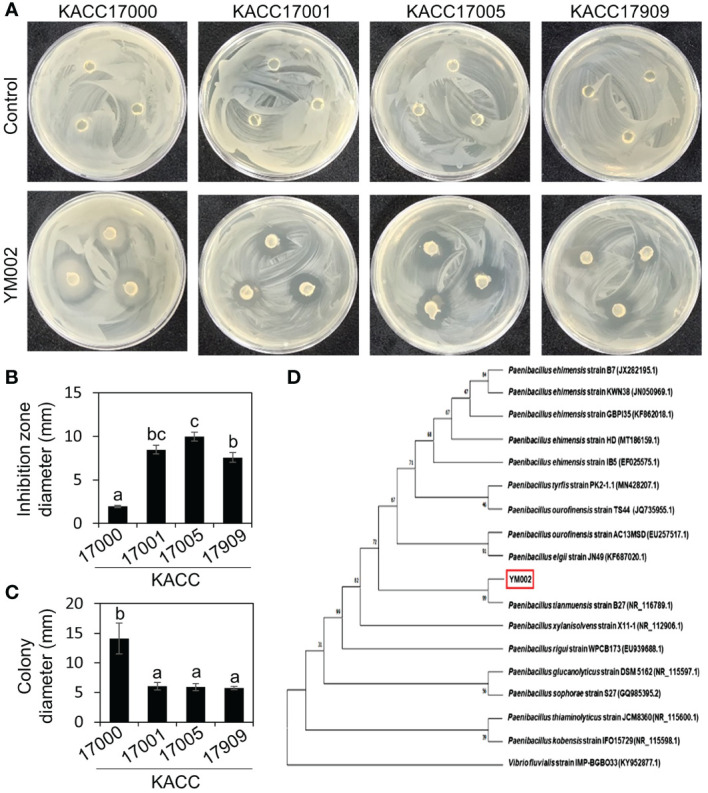
Isolation and identification of antagonistic bacteria *Paenibacillus tianmuensis* strain YM002 (YM002) against different strains of *Acidovorax citrulli.* **(A)** Representative pictures showing the growth inhibition of *A citrulli* around YM002 agar plugs. KACC17000, KACC17001, KACC17005 and KACC17909 are accession numbers of different strains of *Acidovorax citrulli* obtained from Korean Agricultural Culture Collection (KACC; http://genebank.rda.go.kr/). **(B)** Inhibition zone diameter induced by YM002 against different strains of *A citrulli*. The inhibition zone diameter were measured by Image J software. **(C)** Enhanced swimming motility of YM002 in the presence of *A citrulli* KACC17000. Colony diameter of YM002 was measured during the agar plug assay in the presence of different strains of *A citrulli*. Different letters indicated significant statistical differences between treatments (Duncan’s multiple range test; *P* < 0.05). **(D)** Phylogenetic tree analysis of 16S rRNA gene of YM002. MEGAX software was used to construct phylogenetic trees using the maximum-likelihood method. Bootstrap values based on 1,000 replicates were shown at each node. *Vibrio fluvialis* (KY952877.1) was used as an outgroup.

We hypothesized that rhizosphere of various plants growing in mountain area contains useful and distinct microflora, and some of them exhibit antagonistic effect against plant pathogenic bacteria, such as *Acidovorax citrulli*. A total of 240 unknown bacterial strains were isolated from rhizosphere soil samples of various weeds and screened for their antibacterial activity against different strains of *Acidovorax citrulli* (KACC 17000, 17001, 17005, and 17909) in an agar plate assay. Only one unknown bacterial strain showed distinct antagonistic activity against all tested *A. citrulli* strains (**Figure 1A**). This isolate was named YM002 and used for further study. YM002 showed a significant inhibition zone against four different strains of *A. citrulli* on KB agar plates. Inhibition zone diameters induced by YM002 against *A*. *citrulli* strains 17000, 17001, 17005, and 17909 were 1.95 ± 0.13, 8.47 ± 0.49, 9.97 ± 0.5 and 7.57 ± 0.55 mm, respectively (**Figure 1B**). Interestingly, YM002 showed enhanced swimming motility in the presence of *A*. *citrulli* strain 17000 (**Figure 1C**). To identify YM002, the 16s rRNA gene sequence was amplified, sequenced, and subjected to phylogenetic analysis. From the NCBI BLAST (https://blast.ncbi.nlm.nih.gov/Blast.cgi) search, the 16s rRNA gene sequence of YM002 showed the highest sequence similarity with bacteria belonging to the genus *Paenibacillus*. Among different *Paenibacillus* spp. analyzed, YM002 showed the highest homology at 99.0% with *P. tianmuensis* strain B27 (Accession no. NR_116789.1) (**Figure 1D**). Based on the results, YM002 was identified *as P. tianmuensis.*

### 3.2 Biocontrol effect of YM002 against *A*. *citrulli*

**Figure 2:**
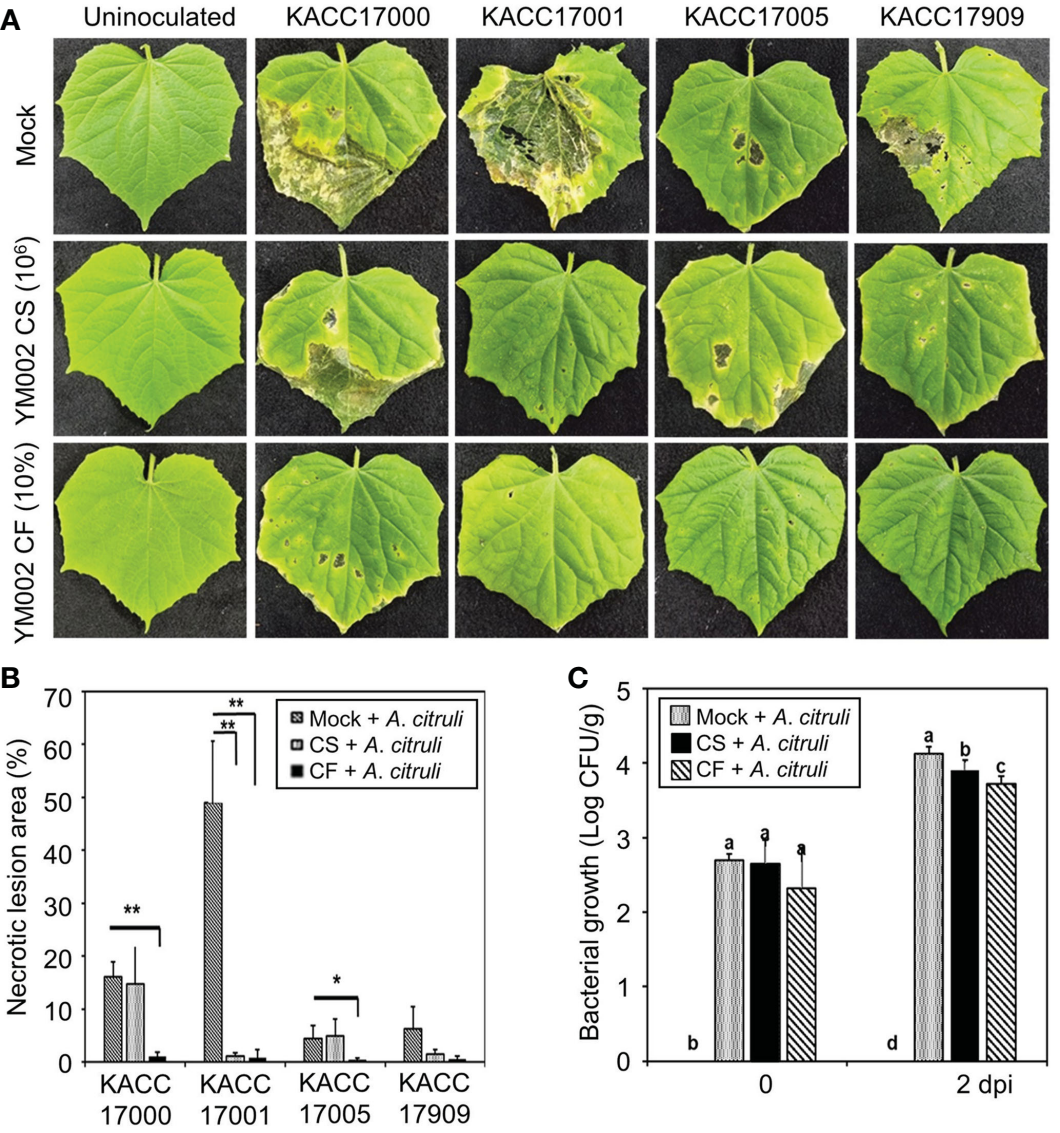
Biocontrol activity of YM002 against *A citrulli* in cucumber plants. **(A)** Represent pictures of cucumber leaves infected with different strains of *A citrulli* with or without YM002 pre-treatment. For cell suspension (CS) or culture filtrate (CF) treatment, 1 × 10^6^ CFU ml^-1^ of YM002 or 10% CF with 0.01% tween 20 were sprayed, respectively, 1 day before *A citrulli* inoculation. **(B)** Necrotic lesion area induced by *A citrulli* with or without pre-treatment with CS or CF of YM002. The necrotic lesion area was measured using Image J software. Data are mean ± standard deviation (n=8). Asterisks indicate significant difference between treatments (*t*-test, *P<0.05, **P<0.005). **(C)** Bacterial growth of *A citrulli* in cucumber leaves with or without pre-treatment with CS or CF of YM002. Experiment were performed with eight replicates. Data are mean ± standard deviation (n=8). Different letters indicated significant differences between treatments (Duncan’s multiple range test; *P* < 0.05).

To test whether YM002 can suppress disease symptoms, fully expended cucumber leaves were spray-inoculated with 4 different strains of *A. citrulli* (1 × 10^7^ cfu/ml) at 24 h after treatment with cell suspension (CS) or culture filtrate (CF) of YM002. As shown in **Figure 2A** (upper panel), all *A. citrulli* strains induced necrotic water-soaking lesions in cucumber leaves at 5 days post inoculation (dpi); however, different strains showed different levels of virulence in cucumber leaves. The *A*. *citrulli* strain KACC17001 showed the highest virulence, and necrotic lesion area of 49.05 ± 12.19% (Mean ± SD) in cucumber leaves (**Figure 2B**). On the other hand, the necrotic lesion area induced by KACC17000, KACC17005 and KACC17909 were 16.11 ± 2.78, 4.50 ± 2.47 and 6.35 ± 4.12% (Mean ± SD), respectively, suggesting KACC17001 is the most virulent strain on tested cucumber plants. Thus we used *A. citrulli* KACC17001 for further experiments. Bacterial growth of *A. citrulli* KACC17001 was measured by using the spontaneous mutant to rifampicin (**Figure 2C**). Treatment of cucumber plants with CS or CF of YM002 significantly reduced growth of *A. citrulli* KACC17001.

**Figure 3:**
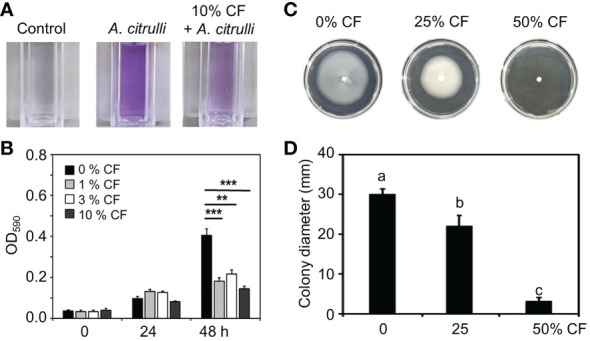
Inhibition of biofilm formation and swimming motility of *A citrulli* strain KACC17001 by CF of YM002. **(A)** Reduced biofilm formation ability of *A citrulli* in the presence of 10% CF of YM002. **(B)** Quantification of biofilm formation level of *A citrulli* in the presence of 0, 1, 3 and 10% CF of YM220. Data are mean ± standard deviation (n=8). Asterisks indicate significant difference between treatments (*t*-test, **P<0.005, ***P<0.0005). **(C)** Reduced swimming motility of *A citrulli* in the presence of different concentrations of CF of YM002. **(D)** Colony dimeter of *A citrulli* in the presence of different concentrations of CF of YM002. Data are mean ± standard deviation (n=8). Different letters indicated significant differences between treatments (Duncan’s multiple range test; *P* < 0.05).

Importantly, cucumber leaves pretreated with either the CS or CF of YM002 showed significantly reduced necrotic symptom area development after the infection with *A*. *citrulli* strains (**Figures 2A, B**). Pretreatment of cucumber leaves with CS of YM002 significantly decreased necrotic lesion area by 97.56% after inoculation with *A*. *citrulli* strain KACC17001 (**Figure 3B**), but not with KACC17000, KACC17005, and KACC17909. Necrotic lesion area in cucumber leaves by KACC17000, KACC17001, and KACC17005 was also significantly decreased by 93.57, 98.34, and 91.27% upon pretreatment with CF of YM002. This suggests that CF of YM002 is more effective than CS of YM002 for controlling the disease. Bacterial growth of *A*. *citrulli* strain KACC17001 was counted from the inoculated cucumber leaves (**Figure 3C**). Both CS and CF of YM002 treatments significantly lowered bacterial growth of *A*.*citrulli* at 2 dpi. Similar to the observed disease severity, CF of YM002 showed a higher inhibitory effect on bacterial growth compared to those of CS of YM002. This suggests CF of YM002 show significant *in vivo* antagonistic activity against *A*. *citrulli*.

### 3.3 Inhibition of biofilm formation and swimming motility of *A*. *citrulli* by YM002

In agar plate assay, YM002 showed growth inhibition activity against all tested A. citrulli strains (**Figures 1A, B**). Thus direct bactericidal/bacteriostatic activity of YM002 was tested by agar well diffusion assay (**Supplementary Figure 1**). Unexpectedly, CF of YM002 did not show bactericidal/bacteriostatic activity against A. citrulli. Thus, we further tested whether CF of YM002 can alter pathogenesis-related phenotypes of A. citrulli. Reportedly, biofilm formation and swimming motility are considered as essential factors in the pathogenesis of A. citrulli (Bahar et al., 2009; Bahar et al., 2010). First, the biofilm formation by A. citrulli strain KACC17001 was measured at OD590nm every 24 h in the presence of 0, 1, 3, and 10% of YM002 culture filtrate (**Figures 3A, B**). Biofilm formation of A. citrulli strain 17001 was significantly decreased by 55.16, 46.36 and 64.27% at 48h after incubation with 1, 3, and 10% CF compared to the negative control, which contained 0% CF (**Figure 3B**). Swimming motility of A.citrulli strain KACC17001 was also measured on a soft nutrient agar (NA) plate supplemented with 0, 25 and 50% of YM002 culture filtrate. The growth diameter was significantly (P < 0.05) decreased by 23.34 and 86.91% in the presence of 25 and 50% of CF of YM002 compared to soft NA plate without CF (**Figures 3C, D**). These findings suggest that CF of YM002 can suppress the biofilm formation and swimming motility of *A*. *citrulli*.

### 3.4 Defense-related gene expression by YM002

**Figure 4:**
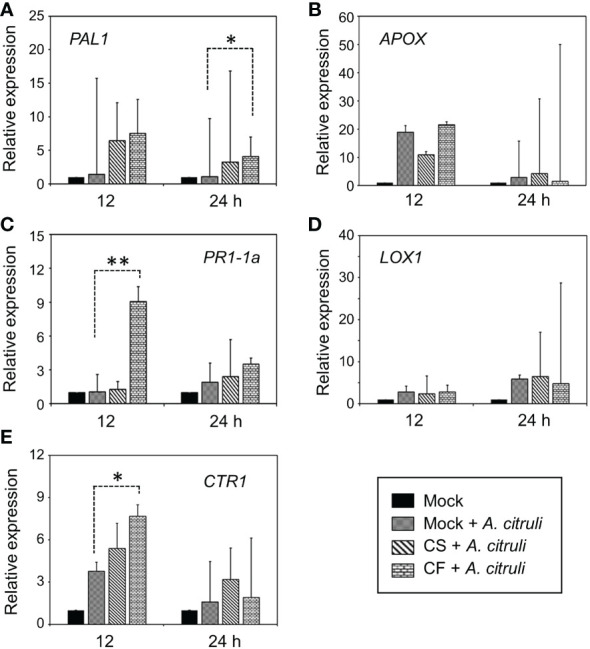
Altered expression patterns of defense-related genes by pre-treatment with CS or CF of YM002. **(A-E)** Relative expression of *PAL1* **(A)**, *APOX* **(B)**, *PR1-1a* **(C)**, *LOX1* **(D)**, *CTR1* **(E)** in cucumber leaves 12 and 24 hpi with *A citrulli* strain KACC17001 with or without pre-treatment with CS or CF of YM002. Data are mean ± standard deviation (n=8). Asterisks indicate significant difference between treatments (*t*-test, *P<0.05, **P<0.005).

To test whether YM002-induced enhanced resistance of cucumber plants could be due to its induced resistance (IR)-inducing activity, the expression of defense-related genes was tested by using the real-time qRT-PCR. For gene expression analysis, two-week-old cucumber leaves were pretreated with either CS (1 × 10^6^ CFU ml^-1^) or 10% CF of YM002 at 24h before the inoculation with *A*. *citrulli* strain KACC17001. Defense-related gene expression levels were measured in cucumber leaves at 12 and 24 hours post inoculation (hpi) with *A*. *citrulli* strain KACC17001 (**Figure 4**). Among the different genes tested, *CONSTITUTIVE TRIPLE RESPONSE 1* (*CTR1*) and *Pathogenesis-related protein 1-1a* (*PR1-1a*) were significantly increased at 12 hpi with *A*. *citrulli* strain KACC17001 by 2.03- and 8.63-fold, respectively, in CF of YM002-treated leaves compared to mock-treated ones. Expression of *Phenylalanine ammonia-lyase 1* (*PAL1*) was significantly increased at 24 hpi with *A*. *citrulli* strain KACC17001 by 3.82-fold in CF of YM002-treated leaves compared to mock-treated ones. On the other hand, expression levels of *Ascorbate peroxidase* (*APOX*) and *Lipoxygenase 1* (*LOX1*) were not significantly different between the treatments.

### 3.5 Plant growth-promoting (PGP) activities of YM002

**Figure 5:**
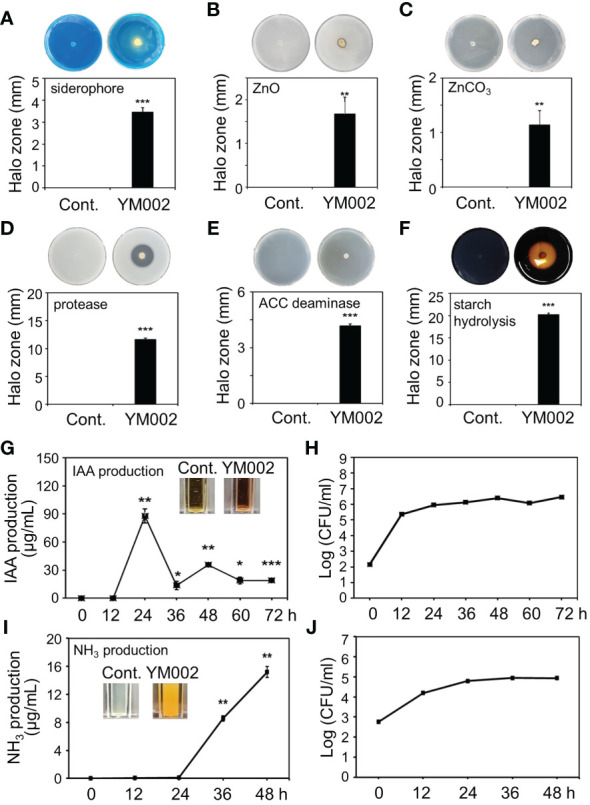
Plant-growth promotion (PGP)-related traits of YM002. **(A-F)** Siderophore production **(A)**, extracellular zinc solubilization **(B, C)**, extracellular protease **(D)**, ACC deaminase production **(E)**, starch hydrolysis **(F)** activities of YM002. **(G-J)** Quantitative indole-3-acetic acid (IAA) **(G)** and NH_3_ **(I)** production by YM002. Bacterial growth was monitored during IAA **(H)** and NH_3_ **(J)** production assays. Data are mean ± standard deviation (n=4). Asterisks indicate significant difference between treatments (*t*-test, *P<0.05, **P<0.005, ***P<0.0005).

Many *Paenibacillus* spp. are known to have different PGP activities (Grady et al., 2016). Thus the different PGP activities of YM002 were tested (**Figure 5**). Notably, YM002 showed different PGP activities. The yellow halo zone surrounding the agar plug of YM002 on CAS agar plates indicates a positive result for siderophore production. The halo zone diameter was 3.46 ± 0.21 mm (Mean ± SD) at 7 dpi (**Figure 5A**). Zinc solubilization activity was observed on YM002-inouclated PKV medium supplemented with zinc oxide (ZnO), and zinc carbonate (ZnCO_3_) (**Figures 5B, C**). Halo zone diameter was measured 1.67 ± 0.39 and 1.13 ± 0.26 mm (Mean ± SD) at 5 dpi, respectively. The extracellular protease production ability of YM002 was confirmed through the clear zone formation on skim milk agar plates (**Figure 5D**). The clear zone diameter induced by YM002 was 11.61 ± 0.26 mm (Mean ± SD) at 5 dpi. YM002 showed successful growth in minimal DF agar plates with ACC as the sole nitrogen source, suggesting it has ACC deaminase activity (**Figure 5E**). The growth diameter of YM002 was 4.18 ± 0.10 mm (Mean ± SD) at 7 dpi. YM002 showed a starch hydrolysis activity as it developed clear zone surrounding the colony at the center of the starch agar plate (**Figure 5F**). YM002-induced halo zone diameter was 20.22 ± 2 mm (Mean ± SD) at 7 dpi. To confirm the IAA production activity of YM002, the supernatant of YM002 was collected up to 72 hpi and reacted with salkowski’s reagent. The color of the mixture was observed positive results development of yellow to pink (**Figure 5G**). Quantitative estimation of IAA production from YM002 revealed that the maximum IAA production of 87.85 ± 7.45 μg/ml (Mean ± SD) was measured at 24 h after incubation. Lower levels of IAA production were observed from 36 to 72 hpi. In this experiment, the bacterial cell number of YM002 was almost saturated within 24 hpi, but its level was maintained up to 72 hpi (**Figure 5H**). The ammonia (NH_3_) production ability of YM002 was also analyzed. Mixing YM002 supernatant with Nessler reagent induced a color change from brown to yellow, suggesting successful generation of ammonia (**Figure 5I**). The production of ammonia was observed initially at 36 hpi, and its level was increased by 15.18 ± 0.77 μg/ml at 48 hpi. Similar to the IAA production assay, the bacterial cell number of YM002 was almost saturated within 24 hpi, but its level was maintained up to 45 hpi (**Figure 5J**). Taken together, YM002 showed various PGP activities, such as siderophore production, zinc solubilization, extracellular protease production, ACC deaminase, starch hydrolysis, IAA production, and NH_3_ production activities.

**Figure 6:**
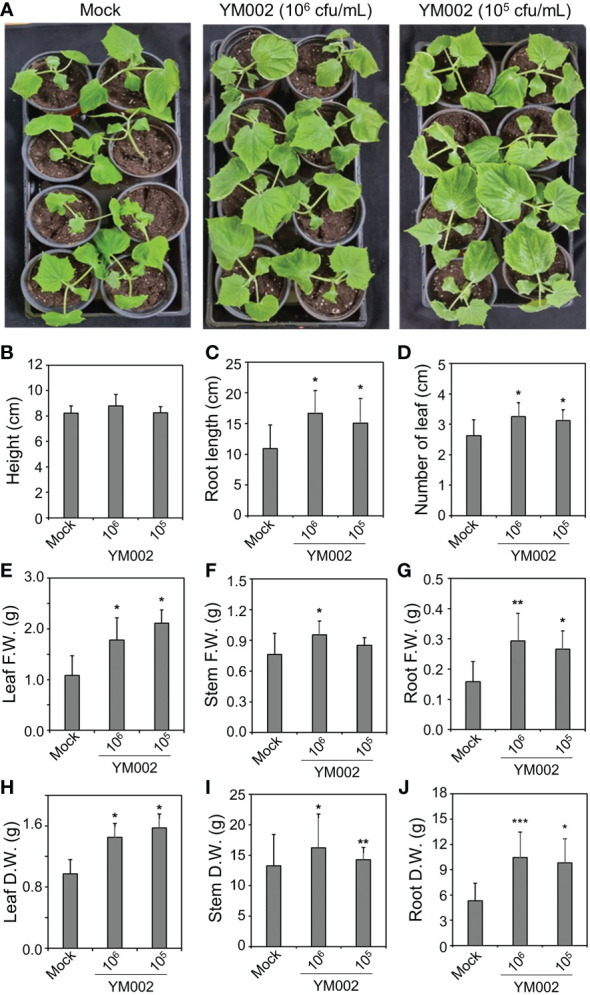
PGP activity of YM002 in cucumber plants **(A)** Enhanced growth of cucumber plants by YM002 treatment. Roots of 2-week-old cucumber plants were submerged into 1 × 10^5^ or 1 × 10^6^ CFU mL^-1^ YM002 cell suspension. Pictures were taken at 14 days after YM002 treatment. **(B-J)** Growth parameters of cucumber plants, including height **(B)**, root length **(C)**, number of leaves **(D)**, leaf fresh weight (F.W.) **(E)**, stem F.W. **(F)**, root F.W. **(G)**, leaf dry weight (D.W.) **(H)**, steam D.W. **(I)** and rood D.W. **(J)** with different concentrations of YM002 treatment. Data are mean ± standard deviation (n=4). Asterisks indicate significant difference between treatments (*t*-test, *P<0.05, **P<0.005, ***P<0.0005).

To test whether the PGP activity of YM002 can actually promote the growth of cucumber plants, two-week-old cucumber seedling plants were inoculated with 1 × 10^5^ and 1 × 10^6^ CFU/ml of YM002. As shown in **Figure 6A**, the cucumber inoculated with CS of YM002 showed enhanced plant growth 14 dpi. Most of the growth parameters, except the height of plants, were significantly enhanced (**Figures 6B–J**). In cucumber plants treated with 1 × 10^6^ and 1 × 10^5^ CFU/ml of YM002, root length (52.58 and 38.03%), number of leaves (23.81 and 19.05%) were significantly increased. In addition, 1 × 10^6^ and 1 × 10^5^ CFU/ml of YM002 treatments also increased fresh weight of leaves (64.48 and 94.69%), stems (25.20 and 18.57%) and roots (85.04 and 67.72%), as well as dry weight of leaves (48.72 and 61.54%), stems (22.1 and 7.5%) and roots (96.93 and 85.38%) compared to mock-treated plants. This suggests that YM002 not only enhances the defense against *A*. *citrulli* but also promote the growth of cucumber plants.

## 4 Discussion

*Acidovorax citrulli* causes BFB mainly in cucurbits, including watermelon, melon, cucumber, squash, pumpkin, and gourds (Schaad et al., 2008). Previous reports suggest that *A*. *citrulli* caused disease in cucumbers under natural conditions, and the typical symptoms were characterized primarily in leaves, but not in plant stems or fruits (Martin et al., 1999). However, inoculation of cucumber fruits by injection with *A*. *citrulli* induced typical BFB symptoms in cucumber fruits (Liu et al., 2012). In this study, the antagonistic activity of *P*. *tianmuensis* strain YM002 against 4 different strains of *A*. *citrulli* was confirmed through dual culture assay. In addition, the pretreatment with cell suspension (CS) or culture filtrate (CF) of YM002 significantly reduced necrotic lesion area and bacterial growth in *A*. *citrulli*-inoculated cucumber plants. Previous studies showed that different microorganisms, such as *Streptomyces* spp., *Pichia anomala*, *Bacillus amyloliquefaciens, Paenibacillus polymyxa*, and *Sinomonas atrocyanea*, showed antagonistic activity against *A*. *citrulli*, and significantly reduce the BFB symptoms in different cucurbits, such as watermelon and melon (Yaeram et al., 2006; Wang et al., 2009; Jiang et al., 2015; Adhikari et al., 2017). To the best of our knowledge, however, the effective antagonistic microorganism is not reported against cucumber BFB. Our study provide the evidence for potential use of *Paenibacillus tianmuensis* strain YM002 as a biological control agent against *A*. *citrulli*, which induces BFB in cucumber.

*Penibacillus* sp. is known to be effective as a biological control agent against various pathogens in plants (Yang et al., 2004; Haggag, 2007; Zhou et al., 2016). *Penibacillus* sp. regulates phytohormones and induces IR, thereby protecting them from potential threats of pathogens (Phi et al., 2010). Furthermore, antimicrobial produced from *Penibacillus* sp. includes peptides, enzymes, and volatile organic compounds, which inflict direct damage against pathogens (Raza et al., 2015). Interestingly, in our experiments, culture filtrate (CF) of YM002 was more effective in reducing the necrotic lesion area and in planta bacterial growth than cell suspension (CS) of YM002, but CF did not show direct bactericidal/bacteriostatic activity in agar diffusion assay. This suggest that YM002 may produce some specific bactericidal/bacteriostatic compounds only in the presence of *A*. *citrulli* [Note that YM002 showed growth inhibition activity against *A*. *citrulli* in agar plate assay, where YM002 and *A*. *citrulli* grow together. However, CF prepared from YM002 culture, which is prepared in the absence of *A*. *citrulli*, did not show bactericidal/bacteriostatic activity]. Thus further studies will be needed to test different media and/or elicitors which can induce the production of specific bactericidal/bacteriostatic compounds from YM002 against *A*. *citrulli*. Different types of secretion systems play a key role in the pathogenesis of *A*. *citrulli* in host plants (Burdman and Walcott, 2012). Among them, Type IV pili is involved in bacterial motility and biofilm formation, allowing it to attach to and/or spread within the host plant. For example, a *pilM* mutant of *A*. *citrulli*, which encodes a protein required for Type IV pili assembly, showed reduced biofilm formation and pathogenicity compared to the wild-type strain (Bahar et al., 2009; Bahar et al., 2010). *A*. *citrulli* M6 *pilT* mutant, an ATPase that is required for IV pili retraction, showed lower biofilm formation, swimming motility, and pathogenicity (Bahar et al., 2009; Bahar et al., 2010). These findings suggest that Type IV pili-mediated biofilm formation and swimming motility contribute significantly to the pathogenicity of *A*. *citrulli*. Importantly, CF of YM002 significantly reduced biofilm formation and swimming motility of *A*. *citrulli*. Thus our findings together with previous report suggest that reduced swimming motility and biofilm formation by CF of YM002 can be a direct mechanism of action of enhanced resistance in cucumber plants against *A*. *citrulli*. In addition, CF of YM002 significantly enhanced the expression of some of the tested defense-related genes, such as *PAL1*, *PR1-1a* and *CTR1*. *PAL1* encodes phenylalanine ammonia lyase, which catalyzes the conversion of L-phenylalanine to ammonia and trans-cinnamic acid. Trans-cinnamic acid is further converted into plant defense-related hormone salicylic acid (SA), which plays a key role in defense against biotrophic and hemibiotrophic pathogens (Klessig et al., 2018). The *PR1-1a* genes encoding PR protein appear to be related to the SA signaling pathway (Sticher et al., 1997). The *CTR1* gene functions as a negative regulator of ethylene (ET) signaling (Huang et al., 2003). During the activation of plant defense response, SA signaling plays a key role against biotrophic and hemibiotrophic pathogens, whereas jasmonic acid (JA)/ET are predominantly involved against necrotrophic pathogens (Koo et al., 2020). Thus the enhanced expression of *CTR1* by CF of YM002 suggest possible suppression of ethylene signaling which is antagonistic against SA signaling. In turn, *CTR1*-induced derepression of SA signaling may contribute to induced resistance against hemibiotrophic pathogen *A*. *citrulli*. These findings suggest that CF of YM002 may enhance the SA-dependent defense signaling pathways during the *A*. *citrulli* infection, thereby enhancing the resistance of cucumber plants. Taken all the available evidence together, CF of YM002 can be useful biocontrol material for cucumber BFB disease, *via* either suppressing the motility and biofilm formation of *A*. *citrulli* or activating SA-dependent defense signaling of cucumber plants.

The genus of the bacterium *Penibacillus* sp. has been isolated from a variety of environments, animals, plants, soils, and humans worldwide. Many *Penibacillus* spp. are known to have PGP effects, including nitrogen fixation (Liu et al., 2019), phosphate solubilization (Wang et al., 2012), iron acquisition (Raza and Shen, 2010; Zhou et al., 2016), and phytohormone production (Lebuhn et al., 1997). As expected, YM002 also exhibited various PGP-related traits, such as siderophore production, zinc solubilization, extracellular protease production, ACC deaminase, starch hydrolysis, IAA production, and NH_3_ production activities. In addition, root-inoculation of cucumber seedling plants with YM002 indeed enhanced various growth parameters. Most of the PGP-related traits of YM002, such as siderophore production, zinc solubilization, extracellular protease production, starch hydrolysis, and NH_3_ production activities, are related with enhance utilization of soil nutrient. Reportedly, ACC deaminase can degrade ACC, a precursor of ET, to ammonia and ketobutyrate (Gamalero and Glick, 2015; Singh et al., 2015). ACC deaminase lowers the ACC level of plants subjected to various abiotic stresses, such as salt stress (Wang et al., 2016) and flood stress (Etesami et al., 2014), and consequently reduces ET-mediated damage to plants. Thus ACC deaminase production by YM002 may also reduce the sensitivity of cucumber plants against different abiotic stresses. Unlike other well-known *Penibacillus* sp., such as *P*. *polymyxa* (Lee et al., 2020; Ali et al., 2021), few studies have been reported on the PGP properties of *P*. *tianmuensis* and its PGP activities in plants. Therefore, our study suggests the potential of *P*. *tianmuensis* not only as a novel biological control agent against *A*. *citrulli*, but also as a novel PGPR of cucumber plants.

Our study indicates that *P*. *tianmuensis* strain YM002 has great potential as a biocontrol for BFB in cucumber by *A*. *citrulli* and as a PGPR. Treatment of cucumber plants with CS or CF of YM002 not only enhanced the growth of cucumber plants, but also enhanced resistance to *A*. *citrulli*. Further experiments will be needed to obtain in-depth information on the mechanism of secondary metabolites of YM002 for its antagonistic effects on *A*. *citrulli*, and its growth promotion activity as well. In addition further safety studies on the use of YM002 will be needed to ensure its safe use in the Agricultural system.

## Data availability statement

The original contributions presented in the study are publicly available. The 16s rRNA sequence data of YM002 can be found here: [https://www.ncbi.nlm.nih.gov/nucleotide/accessionnumber:OQ826657].

## Author contributions

YK and HC planned the projects. YK and HC analyzed data and wrote the manuscript. YK and AY performed the experiments. All authors contributed to the article and approved the submitted version.
