# Supplementary material for: Isolation and Identification Antagonistic Bacterium Paenibacillus tianmuensis YM002 against Acidovorax citrulli
Source: Front Plant Sci. 2023 Jun 12;14:1173695. doi: 10.3389/fpls.2023.1173695 (PMC10292757; doi:10.3389/fpls.2023.1173695)
Supplement: Supplementary file 1 [file DataSheet_1.pdf]

Supplementary Table 1. Defense-related gene primers used for real-time qPCR

Gene	Primer Sequence	Annealing Temp.	Amplicon size	Reference
Actin	F: 5’-ATTGTTCTCAGTGGTGGTTCTAC-3’<br>R: 5’-CCTTTGAGATCCACATCTGCT-3’	58°C	190 b.p.	Song et al., 2018
PR1-1a	F: 5’-GTTGGGCCGATTGAGTGG-3’<br>R: 5’-GCATCTCACTTTGGCACATCC-3’	58°C	285 b.p.	Sang and Kim, 2011
PAL1	F: 5’-ATGGAGGCAACTTCCAAGGA-3’<br>R: 5’-CCATGGCAATCTCAGCACCT-3’	58°C	200 b.p.	Pu et al., 2014
APOX	F: 5’-TCTTGCATGGCACTCTGCTG-3’<br>R: 5’-GCCCTACCCAATGTGTGACCACC-3’	58°C	387 b.p.	Sang and Kim, 2011
CTR1	F: 5’-AAACACGTCGGATAAATATGGCTT-3’<br>R: 5’-CATCCATTCAGGCGTTCCAG-3’	58°C	200 b.p.	Pu et al., 2014
LOX1	F: 5’-AAGGTTTGCCTGTCCCAAGA-3’<br>R: 5’-TGAGTACTGGATTAACTCCAGCCAA-3’	58°C	200 b.p.	Pu et al., 2014

Reference

Pu, X., Xie, B., Li, P., Mao, Z., Ling, J., Shen, H., Zhang, J., Huang, N., Lin, B. (2014). Analysis of the defence-related mechanism in cucumber seedlings in relation to root colonization by nonpathogenic *Fusarium oxysporum* CS-20. *FEMS Microbiol. Lett.* 355(2), 142-51. doi: 10.1111/1574-6968.12461

Sang, M.K., Kim, K.D. (2011) Biocontrol activity and primed systemic resistance by compost water extracts against anthracnoses of pepper and cucumber. *Phytopathology* 101(6), 732-40. doi: 10.1094/PHYTO-10-10-0287

Song, M., Wei, Q., Wang, J., Fu, W., Qin, X., Lu, X., Cheng, F., Yang, K., Zhang, L., Yu, X., Li, J., Chen, J., Lou, Q. (2018). Fine Mapping of *CsVYL*, Conferring Virescent Leaf Through the Regulation of Chloroplast Development in Cucumber. *Front. Plant Sci.* 9, 432. doi: 10.3389/fpls.2018.00432
