# Supplementary material for: Isolation and Identification Antagonistic Bacterium Paenibacillus tianmuensis YM002 against Acidovorax citrulli
Source: Front Plant Sci. 2023 Jun 12;14:1173695. doi: 10.3389/fpls.2023.1173695 (PMC10292757; doi:10.3389/fpls.2023.1173695)
Supplement: Supplementary file 2 [file DataSheet_2.pdf]

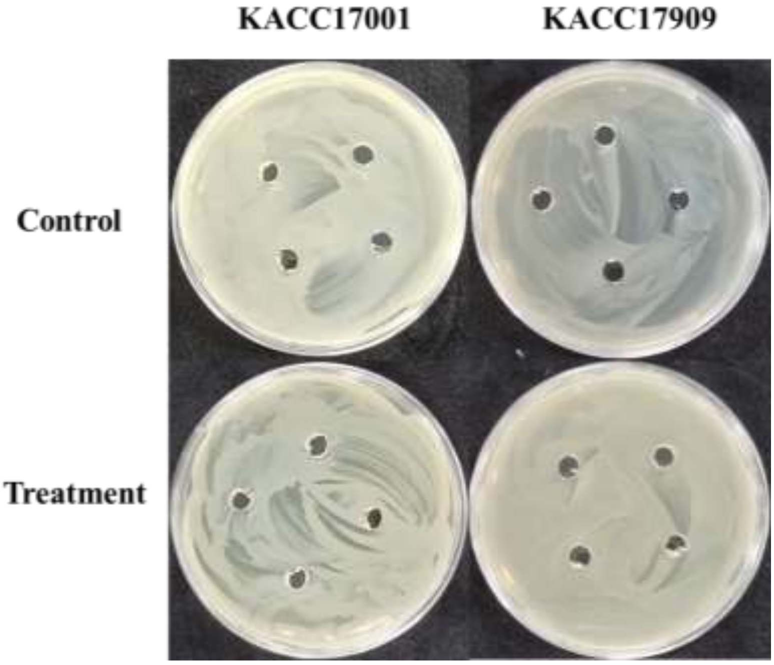

Supplementary Figure 1. No bactericidal/bacteriostatic activity of culture filtrate (CF) of YM002 against *Acidovorax citrulli* strains KACC17001 and KACC17909. Bactericidal/ bacteriostatic activity of CF of YM002 was tested by agar well diffusion assay. The wells were prepared by punching King's B (KB) agar plates seeded with *A. citrulli* by using a sterile cork borer (5.5 mm in diameter). Each wells were filled up with either 50 µL of KB media (Control) or 50 µL of CF of YM002 (Treatment), then incubated at 28℃ for 72h. CF of YM002 was prepared as described in materials and methods section (See 2.5 Biofilm formation assay). CF of YM002 did not show growth inhibition activity against *A. citrulli* strains KACC17001 and KACC17909. Experiments were repeated 3 times with similar results.
